# Carcinoma Cutis

**Published:** 1907-06

**Authors:** John V. Shoemaker

**Affiliations:** Philadelphia; Professor of Materia Medica, Therapeutics, Clinical Medicine and Diseases of the Skin in the Medico-Chirurgical College and Hospital of Philadelphia, 1519 Walnut Street


### Carcinoma Cutis.

### A Clinical Lecture in Abstract.

By JOHN V. SHOEMAKER, M.D., LL.D., Philadelphia.

Professor of Materia Medica, Therapeutics, Clinical Medicine and Diseases of the Skin in the Medico-Chirurgical College and Hospital of Philadelphia,

THIS male patient, age 56 years, nativity Ireland, is suffering from a disease which is typical, yet if not properly studied may lead to making a wrong diagnosis. Over the right mastoid is situated an ulcer the edges of which are everted; the surface is sloughing and the tissues around the ulcer are infiltrated and hard. The patient stated that the ulcer has been increasing in size gradually, is painful at times, with a burning sensation radiating down the side of his neck into his ear. The lymphatic glands in the cervical and submaxillary regions are all hard and enlarged. The skin over the neck and face is covered with many little nodules and areas of carcinomatous tissue. Some of the lesions are large and dark in appearance, while others are smaller and appear to be in a stage of active inflammation. The ulcer over the mastoid first began as a papule, following a slight cut with the razor. Later a crust formed, which came away leaving a raw ulceration.

The ulceration in these cases is due to the increasing pressure of the new material upon the cutaneous vessels, the vascular supply being diminished or cut off from the affected parts and ulceration or gangrene resulting. Fresh nodules develop as a rule in other portions of the body, as seen on this patient's face and neck. The lymphatic glands become hard, nutrition being interfered with, the patient becoming more and more emaciated, and finally perishes from exhaustion.

From the history of the onset, the glandular involvement, and the many nodules on the surrounding portions of the skin, this case can readily and properly be diagnosticated carcinoma cutis. The only diseases with which carcinoma could be confounded are sarcoma, and lymphadenoma. Sarcoma, however, never attacks the lypmhatic glands, while carcinoma even at an early date invades these structures. Lymphadenoma, on the other hand, involves the lymphatic system also, but it is a painless disease of long duration, and usually makes its appearance in the cervical glands. Carcinoma, on the contrary, is of comparatively short duration, is accompanied by more or less pain and by marked emaciation, and the cervical glands are not so much enlarged as in lymphadenoma.

The pathological condition of this form of carcinoma presented under the microscope is a dense network of connective tissue arranged in alveolar masses, the meshes of which are filled with many round epithelial cells.

The etiological factors of carcinoma are still involved in mystery. The most plausible theory of its production is that which supposes it to be due to a disturbance of the functions of the trophic system. Probably it is a chemical change that takes place in the tissues.

The *x*-ray treatment is the best and probably the most available for this patient. I have seen excellent results from the application of the *x*-ray and it may not only heal the ulceration but, as it often has, cause the disappearance of the nodules entirely.

I am in hopes by keeping up the patient's general health with tonics and especially so by giving arsenic, that we may be able to retard the disease and hold it in subjection. The *x*-ray may, in the meanwhile, heal the ulcer which is now in an active stage of ulceration, and also compel the nodules to actually disappear. By this combined treatment we may be able to clear up the deposits upon and in the skin, and in this way prolong the life of the patient.

The prognosis, however, is always unfavorable; few recover from the disease; the majority of the patients die in the course of a few years.

1519 Walnut Street.

